# Iron phosphide anchored nanoporous carbon as an efficient electrode for supercapacitors and the oxygen reduction reaction†

**DOI:** 10.1039/c9ra04326h

**Published:** 2019-08-13

**Authors:** Ahmed K. Yousef, Yena Kim, Piyali Bhanja, Peng Mei, Malay Pramanik, M. M. S. Sanad, M. M. Rashad, A. Y. El-Sayed, Abdulmohsen Ali Alshehri, Yousef Gamaan Alghamdi, Khalid Ahmed Alzahrani, Yusuke Ide, Jianjian Lin, Yusuke Yamauchi

**Affiliations:** Chemistry Department, Faculty of Science, Al-Azhar University Assiut Branch 71524 Assiut Egypt; Key Laboratory of Eco-Chemical Engineering, College of Chemistry and Molecular Engineering, Qingdao University of Science and Technology Qingdao 266042 China; International Center for Materials Nanoarchitectonics (WPI-MANA), National Institute for Materials Science (NIMS) 1-1 Namiki Tsukuba Ibaraki 305-0044 Japan piyalibhanja4@gmail.com yenakim1227@gmail.com; School of Chemistry and Materials Science, South-Central University for Nationalities Wuhan 430074 China; Central Metallurgical Research & Development Institute P.O. Box 87 Helwan 11421 Egypt; Department of Chemistry, King Abdulaziz University P.O. Box. 80203 Jeddah 21589 Saudi Arabia; School of Chemical Engineering, Australian Institute for Bioengineering and Nanotechnology (AIBN), The University of Queensland Brisbane QLD 4072 Australia y.yamauchi@uq.edu.au; Department of Plant & Environmental New Resources, Kyung Hee University 1732 Deogyeong-daero, Giheung-gu Yongin-si Gyeonggi-do 446-701 South Korea

## Abstract

Inspired by their distinctive properties, transition metal phosphides have gained immense attention as promising electrode materials for energy storage and conversion applications. The introduction of a safe and large-scale method of synthesizing a composite of these materials with carbon is of great significance in the fields of electrochemical and materials sciences. In the current effort, we successfully synthesize an iron phosphide/carbon (FeP/C) with a high specific surface area by the pyrolysis of the gel resulting from the hydrothermal treatment of an iron nitrate–phytic acid mixed solution. In comparison with the blank (P/C), the as-synthesized FeP/C appears to be an efficient electrode material for supercapacitor as well as oxygen reduction reaction (ORR) applications in an alkaline medium in a three-electrode system. In the study of supercapacitors, FeP/C shows areal capacitance of 313 mF cm^−2^ at 1.2 mA cm^−2^ while retaining 95% of its initial capacitance value after 10 000 cycles, while in the ORR, the synthesized material exhibits high electrocatalytic activity with an onset potential of *ca.* 0.86 V *vs.* RHE through the preferred four-electron pathway and less than 6% H_2_O_2_ production calculated in the potential range of 0.0–0.7 V *vs.* RHE. The stability is found to be better than those of the benchmark Pt/C (20 wt%) catalyst.

## Abstract 2

Synthesis of a nanoporous FeP/C material through a two-step method involving hydrothermal and carbonization processes for supercapacitors and the oxygen reduction reaction.

## Graphical abstract

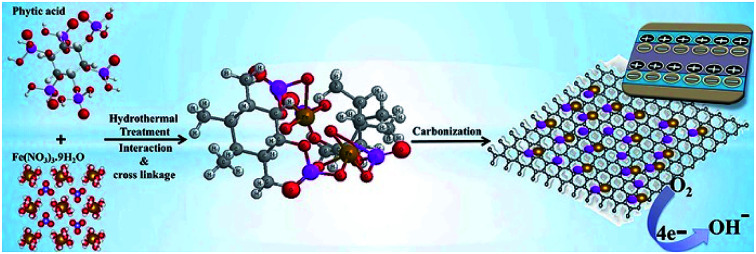

## Introduction

Searching for a new renewable, low-cost, and environmentally friendly source for energy storage and/or conversion has become one of the hottest research challenges, driven by diminishing fossil fuel supplies and climate change in addition to ever-increasing demands for energy sources.^1,2^ Thus, supercapacitors and oxygen reduction reaction (ORR) have been considered the best way out for the current and near future energy issues.^3^ Supercapacitor devices can be made using non-toxic materials and show characteristic properties such as a superior power density, long cycle life, and high charge–discharge efficiency as compared with conventional batteries.^4–6^ The ORR is crucial in our daily life processes, and it is one of the most important reactions in energy storage and conversion applications, where it is the heart of metal–air batteries and fuel cells.^7–9^

The electrical energy in supercapacitors can be stored on the electrode surface in two ways. First, the electrolyte ions are adsorbed at the electrical double layer to produce what is called electric double layer capacitors (EDLCs), and a second one is by redox reactions, which are called pseudocapacitors.^10–12^ Depending on the nature of the electrode materials, the electrical energy storage mechanism could be controlled. Metal oxides and conductive polymers are the main materials used for pseudocapacitors, while carbon is the conventional electrode material used for EDLCs.^13^ In general, a high-performance supercapacitor requires materials with high electrical conductivity and a large ion-accessible surface area. In addition, a high ionic transport rate requires short ion diffusion length throughout the electrode materials.^14^ On the other hand, ORR reacts sluggishly, and it needs to be facilitated to improve its efficiency. Although Pt is the current state-of-the-art electrocatalyst for this reaction, intensive research to modify or replace it is ongoing owing to its high cost and finite resource.^15–17^

In spite of the recently obtained performance of both supercapacitors and ORR, with the rapid development of electronic devices and the extension of their applications toward large-scale devices, much higher performance is required through the improvement of properties such as energy density, rate capability, cyclability, and safety. The development of new electrode materials plays a vital role in reaching the required performance to promote the commercialization of the two applications mentioned above.^18–21^ Transition metal compounds are an important class of functional electrode materials that have been widely investigated in several energy applications.^22–25^ In an inert atmosphere, transition metals combine with phosphorus chemicals to form transition metal phosphides (TMPs), and these materials have notable potential toward energy storage and electrocatalytic applications due to their advantages of low cost, easy large-scale synthesis, and interesting electrical properties.^26^ Among TMPs, iron phosphide (FeP) has been considered as a promising energy electrode material because Fe is naturally abundant and has low cost, low toxicity, and negligible environmental impact.^27^ However, some synthetic methods for producing this kind of material are dangerous (for example, the use of H_2_ to produce PH_3_ gas).^26,28^ Moreover, the poor stability of iron-based materials limits their electrochemical properties.^29–32^ Thus, its combination with carbon is an effective strategy for keeping structural stability, improving electronic conductivity during cycling, and obtaining high surface area materials.^33–35^ Accordingly, providing a safe synthetic method to obtain FeP/carbon (FeP/C) materials with high surface areas and high stability while exploring their performance as supercapacitors and ORR are highly important for satisfying the current needs for energy.

In this study, we report a facile and safe method for the synthesis of FeP/C with a high surface area through a pyrolysis strategy for the gel resulting from the hydrothermal treatment of Fe nitrate–phytic acid mixed solution. When the as-synthesized FeP/C is used as an electrode for a supercapacitor, it exhibits good areal capacitance and shows outstanding cycle performance (retains 95% of its initial capacitance value after 10 000 cycles), which indicates the promising practical applications of synthesized FeP/C for supercapacitors. The electrocatalytic performance towards ORR in an alkaline medium is also investigated. The obtained FeP/C electrodes show electrocatalytic activity through a four-electron reaction pathway that was higher than that of P/C samples. In addition, the limiting current density at 0 V *vs*. RHE and stability are found to be better than those of the benchmark Pt/C (20 wt%) catalyst.

## Experimental section

### Materials

Iron nitrate nonahydrate (Fe (NO_3_)_3_·9H_2_O), isopropanol, ethanol, methanol, and KOH were purchased from Wako Pure Chemicals Co., Ltd. Phytic acid solution (50 wt% in H_2_O) and Nafion solution (5 wt%) were purchased from Sigma-Aldrich, and commercial Pt/C (20 wt%) from Alfa Aesar. Carbon Paper TGP-H-090 was obtained from Toray. All chemicals were of analytical grade and were used without further treatment.

### Synthesis of FeP/C and P/C

In a typical synthesis procedure, 0.5 mmol of Fe (NO_3_)_3_·9H_2_O was added to 10 mmol of phytic acid solution, and it was continuously stirred for one hour. The resultant clear solution was transferred to a 25 mL stainless steel autoclave and heated to 140 °C at a rate of 1 °C min^−1^ and left for 36 h. The resulting gel was transferred to a tubular furnace and left in a nitrogen atmosphere at room temperature for 1 h; after that, the temperature was increased to 300 °C and maintained for 2 h. Subsequently, the temperature was increased to 900 °C and maintained for 2 h. Finally, the sample was naturally cooled. The heating rate for the entire annealing process was 5 °C min^−1^. After cooling to room temperature, a black powder material of FeP/C was obtained. P/C was prepared through the same procedure without adding Fe salt with a slight modification, where the gel resulting from the hydrothermal treatment was washed once with methanol and dried at 60 °C for one day before annealing.

### Characterization

Wide-angle powder X-ray diffraction (XRD) patterns were collected on a Rigaku SmartLab X-ray diffractometer using Cu Kα radiation (40 kV and 30 mA) at a scanning rate of 1° min^−1^. Raman spectroscopy was acquired with a HORIBA Scientific Lab RAM HR Raman spectrometer system using 532.4 nm laser excitation. Field-emission SEM measurements were performed on a HITACHI SU-8230 to check the morphologies. Investigation of the inner structure and energy-dispersive X-ray spectroscopy (EDS) elemental mapping were carried out using a transmission electron microscope (TEM, JEOL JEM-2100F). The nitrogen adsorption–desorption of the samples was measured on a Micromeritics BK122T-B analyzer. The Brunauer–Emmett–Teller (BET) theory was used for the specific surface area (SSA) determination in the relative pressure range of 0.05–0.30. Pore size distributions were determined from the adsorption isotherms, according to the quenched solid-state density functional theory (QSDFT) assuming a mixed cylinder/sphere pore model. Inductively coupled plasma optical emission spectroscopy (ICP-OES) was done on a Hitachi High-Tech Science Corporation model SPS3520UV-DD. The standard was made by Kanto Chemical Co., Inc., and the qualified standard solution by JCSS. The samples were fused by a mixture of Na_2_CO_3_, H_3_BO_3_, KNO_3_ and K_2_S_2_O_7_ salts to digest the material into a dissoluble solid. During this process carbon burns out and removed as CO_2_, Remaining solid melt was then dissolved in HNO_3_ for the chemical analysis.

### Electrochemical measurements

The electrochemical performances of the prepared electrodes were investigated with a three-electrode system.

#### For supercapacitor measurements

Homogeneous ink was prepared as follows. First, the as-prepared FeP/C and P/C were ground, and then 4.0 mg of solid material was dispersed into 380 μL of an isopropanol/water mixed solution (volume ratio of 2 : 1) and 20 μL of 5.0 wt% Nafion. After 30 min sonication, 120 μL of the suspension was dropped onto carbon paper (thickness: 1 mm) with an area of 1 × 1 cm^2^ and dried overnight at 60 °C. The mass loading was 1.2 mg cm^−2^. All electrochemical measurements were carried out using a CHI 660E instrument; cyclic voltammetry (CV) and galvanostatic charge–discharge (GCD) measurements were carried out in 3 M KOH between −0.8 and 0.0 V *vs.* SCE, with a platinum wire and saturated calomel electrode (SCE) as a counter and a reference electrode, respectively. The test of long-term stability for FeP/C was conducted by cycling between −0.8 and 0.0 V *vs.* SCE in 3 M KOH at a scan rate of 100 mV s^−1^.

The gravimetric specific capacitances (*C*_g_, F g^−1^) of the as-synthesized electrodes were calculated from the GCD curves using the following equation:*C*_g_ = (*I* × Δ*t*)/(*m* × Δ*V*),where *I* is the discharge current (A), Δ*t* is the discharge time difference (s), *m* is the mass of the active material (g), and Δ*V* is the potential change during the discharge process (V).

The areal capacitance (*C*_A_, mF cm^−2^) was calculated using the following equation:*C*_A_ = *C*_g_ × *m*_l_,where *m*_l_ is the mass loading of the sample (mg cm^−2^).

#### For ORR measurements

First, the homogeneous ink was prepared as follows. The as-prepared FeP/C and P/C were ground, and then 5.0 mg of the solid material was dispersed into 950 μL of an ethanol : water mixed solution (volume ratio of 1 : 3) and 50 μL of 5.0 wt% Nafion. After sonication for 60 min, 5 μL of the suspension was dropped onto a glassy carbon electrode (GCE) of a rotating ring-disk electrode (RRDE) with an area of 0.1256 cm^2^ and dried under a lamp. The mass loading was 0.2 mg cm^−2^. To activate the GCE, it was pre-polished with 1 μm diamond, then 0.05 μm alumina powder, and washed with water. All electrochemical measurements were carried out using CHI 842B instrument; cyclic voltammetry (CV), linear sweep voltammetry (LSV) at 10 mV s^−1^, and chronoamperometry (*i*–*t*) measurements were performed in 0.1 M KOH saturated solution. The electrolyte solution was continuously purged with ultra-pure O_2_ or N_2_ for at least 1 h before starting the measurements. A platinum wire and silver–silver chloride (Ag/AgCl) were used as the counter and reference electrode, respectively. The potentials were expressed with regard to the reversible hydrogen potential electrode (RHE). The overall electron transfer numbers per oxygen molecule were calculated from the slope of the Koutecky–Levich (K–L) plots according to the following equation:*J*^−1^ = *J*_k_^−1^ + *J*_L_^−1^ = *J*_k_^−1^ + *B*^−1^*ω*^−1/2^*B* = 0.2*nFC*_o_(*D*_o_)^2/3^*ν*^−1/6^,where *J* is the current density, *J*_k_ is the kinetic current density, *J*_L_ is the diffusion-limited current density, *n* is the transferred electron number, *F* is the Faraday constant (*F* = 96 485 C mol^−1^), *C*_o_ is the concentration of O_2_ (1.2 × 10^−6^ mol cm^−3^), *D*_o_ is the diffusion coefficient of O_2_ (1.9 × 10^−5^ cm^2^ s^−1^), *ν* is the kinematic viscosity of the electrolyte (0.01 cm^2^ s^−1^), and *ω* is the electrode rotating speed in rpm (0.2 is a constant when the rotating speed is expressed in rpm).^36,37^*n* and % H_2_O_2_ were calculated from RRDE measurements according to the following equations:*n* = 4 × *I*_d_/[*I*_d_ + (*I*_r_/*N*)]*I*_d_ is the disk current, *I*_r_ is the ring current, and *N* is the collection efficiency of the ring (0.37).% H_2_O_2_ = 200 × (*I*_r_/*N*)/[*I*_d_ + (*I*_r_/*N*)].

## Results and discussion

FeP/C was synthesized through a two-step process involving hydrothermal solution-phase crystallization and carbonization under inert atmosphere. During the crystallization process, the clear mixtures of the phytic acid and Fe(iii) precursor solutions undergo polycondensation from the initial gel through the hydrothermal treatment at 140 °C for 36 h. Under these conditions, the phytic acid molecule, which bears six phosphonic acid moieties, has a strong affinity to bind with Fe(iii) species present in the synthesis medium to form a highly cross-linked structure *via* the Fe–O–P bonds. The cross-linking increased during the hydrothermal treatment, leading to improved mechanical strength of the metal-ligand network structure.^38^ The multidentate nature of the phytic acid molecule together with the presence of PO_4_ tetrahedral and FeO_6_ octahedral units separated by organic fragments could provide high structural robustness, as reflected in the crystalline nanostructure. Finally, the pyrolysis of the resulted cross-linked structure at a high temperature leads to the decomposition of Fe–phytate and the interaction of Fe with P to form FeP nanoclusters, while the phytate rings carbonized to obtain FeP/C as a final product.

**Fig. 1:**
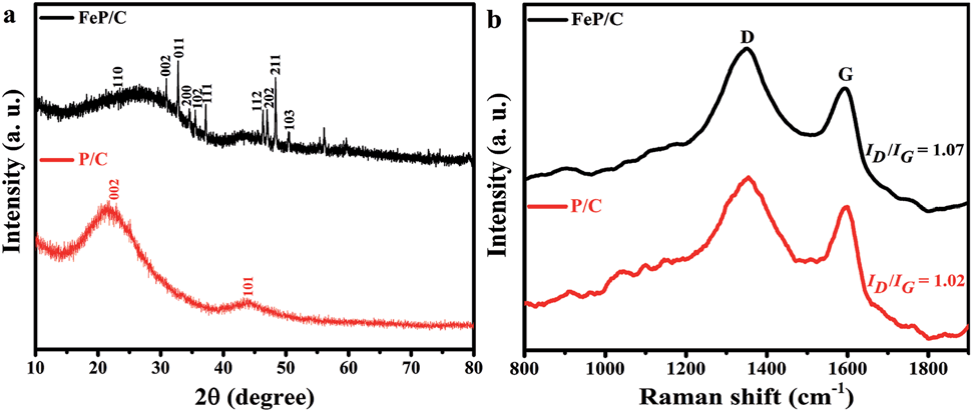
(a) XRD patterns and (b) Raman spectra of P/C and FeP/C.

The XRD patterns of P/C and FeP/C are shown in Fig. 1a. All XRD peaks in the FeP/C sample can be indexed as pure FeP (JCPDS card no. 89-2746) with the presence of two broad characteristic graphitic peaks located at around 23° and 43° in both samples, which can be assigned to (002) and (101) planes, respectively. Raman analysis was used to investigate the graphitic degree of the carbon content in the synthesized samples. As can be seen in Fig. 1b, both samples exhibit typical D and G bands at 1353 and 1596 cm^−1^, representing the disordered and graphitic phases in carbon, respectively. Therefore, the integrated intensity ratio of D and G bands can reveal the level of ordering and defects in the carbon structure, indicating a higher disorder for a higher *I*_D_/*I*_G_ value.^39^*I*_D_ is higher than *I*_G_ in the two samples, and the intensity ratio of *I*_D_/*I*_G_ is about 1.02 and 1.07 for P/C and FeP/C, respectively. This indicates that many defects or disordered sites are present in the carbon component, which agrees with the obtained XRD data.

**Fig. 2:**
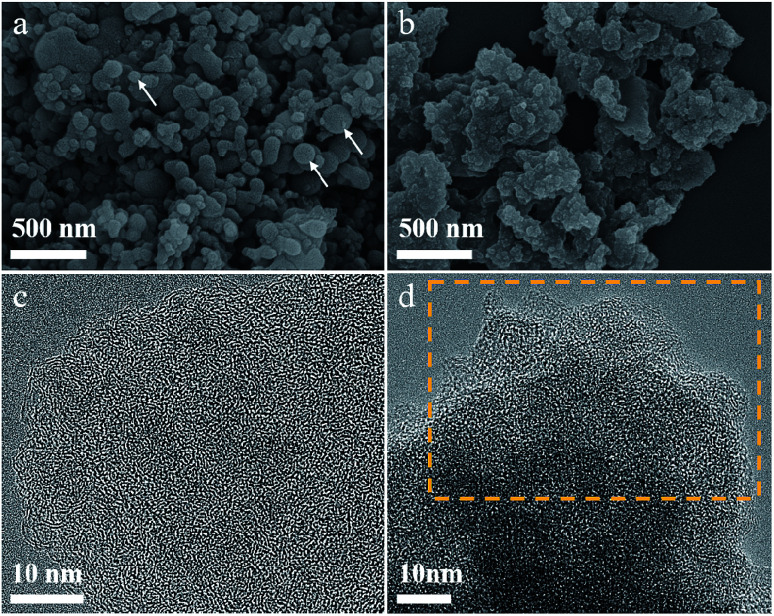
(a and b) SEM and (c and d) HRTEM images of (a and c) P/C and (b and d) FeP/C, respectively. The white arrows in (a) indicate the presence of spherical particles in the P/C sample. The yellow box in (d) indicates the layered-like structure of FeP/C.

The morphology of the P/C and FeP/C materials were investigated using scanning electron microscopy (SEM) (Fig. 2a and b). As shown in Fig. 2a, the P/C possesses irregularly shaped particles, with the presence of some spherical particles (as indicated by arrows). However, the FeP/C appears as a stacked layered structure with no distinct particles, as can be seen in its SEM image (Fig. 2b and S2b†). Thus, the morphology has been changed from P/C to FeP/C, which is understandable where the temperature is high enough to cause a reaction of Fe particles with the carbon matrix to form Fe carbide, which, with increasing temperature, dissociates again into graphitic carbon and Fe particles.^40^ A variety of graphitic carbons of small crystallite size are supposed to be formed, as the graphitization effect of the Fe particle is localized. The high temperature (900 °C) and the presence of the Fe particle enhance the degree of graphitization.^41^ The amorphous nature of the synthesized materials and the disordered micropores were observed under a high-resolution transmission electron microscope (HRTEM), as shown in Fig. 2c, d and S1.† The HRTEM images indicate the layered-like structure of synthesized FeP/C samples, highlighted by the yellow boxes in Fig. 2d and S2.† This sheet-like structure can improve the electrocatalytic performance of active materials through fast electron transfer and ion diffusion pathways, and can also increase the effective contact area between the electrolytes and active materials.^42,43^ Elemental mapping of FeP/C shows the uniform distribution of Fe and P elements over the carbon matrix, as can be seen in Fig. S3.† From this elemental mapping, it is noticed that the amount of P element is higher than that of Fe, which is different from the results obtained by XRD (1 : 1). From ICP analysis, the amount of P in the P/C sample was found to be 20%, while in the FeP/C sample, the amount of P was 15% and of Fe was 7%, indicating the dominant content of C in both samples (80%). That may explain its amorphous appearance, as shown in Fig. 2c and d.

**Fig. 3:**
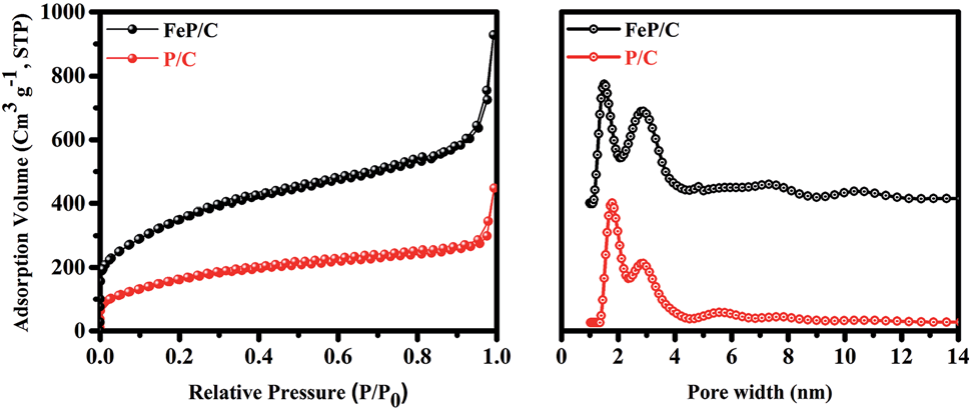
N_2_ adsorption–desorption isotherms and pore size distribution curves of P/C and FeP/C.

The specific surface area and pore size of P/C and FeP/C were examined by nitrogen adsorption–desorption measurements (Fig. 3). Table S1† summarizes the pore sizes, specific surface areas (SSAs), and total pore volumes (TPVs) of both samples. P/C has possessed a high BET surface area of 596 m^2^ g^−1^; after introducing Fe in the synthesis process, it is dramatically increased to 1269 m^2^ g^−1^. The high surface area without the use of any template may be due to the pore-generating ability of the starting precursor. At a sufficiently high temperature, phytic acid releases its six molecules of orthophosphate, which are removed from the surface of carbon as CO_2_ gas and generate mainly micropores in the carbon framework.^44,45^ The higher surface area of FeP/C can be ascribed to its higher total pore volume. Its pore size distribution (Fig. 3b) indicates the micro-/meso-porous structure of both samples. While FeP/C shows a sharp and strong peak at a pore diameter of 1.5 nm, P/C shows a similar peak at 1.8 nm. Both samples show a peak around 2.9 nm. It is confirmed that the FeP/C is highly porous in nature with a high surface area. The large surface area and abundant micro/meso-pores provide sufficient channels for the efficient diffusion of the electrolyte and facilitate the accumulation of electrons on the electrode surface in the electrochemical system. This is expected to achieve stable and high performance as electrode materials for supercapacitor and ORR applications.^46,47^

**Fig. 4:**
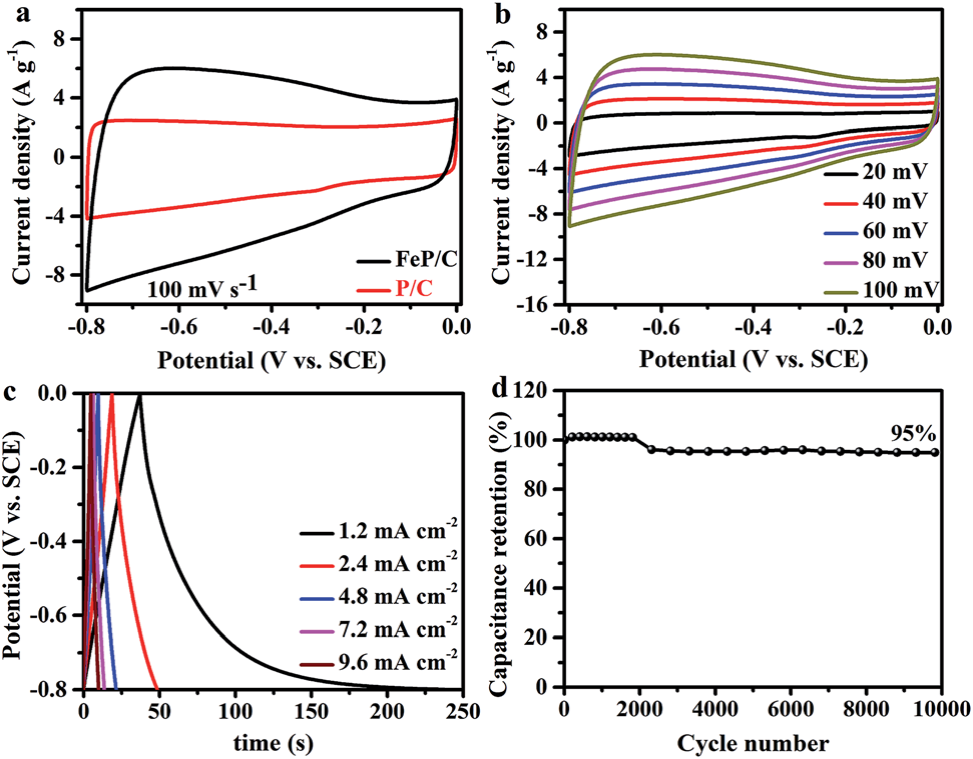
Electrochemical properties measured in a three-electrode system using 3 M KOH as an electrolyte. (a) CV curve comparison of P/C and FeP/C at 100 mV s^−1^ scan rate, (b) CV curves of FeP/C at various scan rates, (c) GCD curves of FeP/C at different current density, and (d) the cycling performance of FeP/C at 100 mV s^−1^.

The supercapacitor (SC) performances of P/C and FeP/C samples were investigated by cyclic voltammetry (CV) and galvanostatic charge/discharge (GCD) measurements in a conventional three-electrode configuration at room temperature using 3 M KOH solution as an electrolyte. Fig. 4a shows a comparison of the CV curves of P/C and FeP/C obtained at a scan rate of 100 mV s^−1^. Both samples show a quasi-rectangular shape, indicating an electric double layer capacitive behavior. The FeP/C electrode shows a considerably higher current density than that of P/C, revealing the higher capacitance of FeP/C based on the directly proportional relationship between the CV curve area and capacitance. The CV curves were also obtained by varying the scan rate for both samples, P/C (Fig. S4a†) and FeP/C (Fig. 4b). The good symmetry shown indicates a good rate capability.

The GCD curves of P/C and FeP/C are depicted in Fig. S4b† and 4c, respectively. Curves of P/C exhibit nearly triangular traces at various current densities, showing the capacitive behavior of the prepared sample. In contrast, the discharging curve in the FeP/C sample shows an initially sharp potential drop, especially at the current density of 1.2 mA cm^−2^, which returns back to the internal resistance (ohmic resistance). This IR drop mainly arises from the internal resistance of FeP/C due to the migration of ions between the electrode and electrolyte.^48^ Fig. S5† shows the change of areal capacitance with current density for both samples. The P/C shows better rate capability than that of FeP/C. This can be understood in the light of the pore size of both samples (Table S1†), where the reversible adsorption/desorption of electrolyte ions on the surface of electrodes determines the electric double layer (EDL) capacitance. At low current densities, electrolyte ions can easily access the inner micro- and meso-pores through holes on the P/C and FeP/C. However, at high current densities, the small pores may reduce the ions' movements. Therefore, the capacitance decreases significantly in the FeP/C sample.^49^

The areal capacitances (*C*_A_) of both electrodes were estimated based on the galvanostatic discharge curves. The *C*_A_ values of P/C and FeP/C achieve 41 and 313 mF cm^−2^ at a current density of 1.2 mA cm^−2^, respectively. The specific capacitance values of P/C and FeP/C at an equivalent current density of 1 A g^−1^ correspond to 34 and 261 F g^−1^, respectively. The obtained areal capacitance of FeP/C is close to or considerably better than some previously reported iron-based materials. Table S2† shows data comparing its electrochemical performance with those of some previously reported Fe-based materials.

The electrochemical stability of the electrode material was evaluated using long-term cycling performance at a scan rate of 100 mV s^−1^ for 10 000 cycles (Fig. 4d). It is well known that because of structural deformation during the long-time charge/discharge process, iron-based supercapacitor electrode materials often suffer from poor stability.^50,51^ In previous studies, arrays from FeP nanotubes and nanorods were used as negative electrodes for supercapacitor application and showed only 41% and 24.19% retention after 5000 cycles, respectively.^29,31^ After coating FeP nanorod arrays with PEDOT, the stability increased to 82.1%.^31^ Porous N/P co-doped carbon showed capacitance retention of 86.3% after 10 000 cycles.^52^ Interestingly, FeP/C exhibits outstanding cycle performance and retains 95% of its initial capacitance value after 10 000 cycles, indicating the synergetic effect of the coupling of FeP and carbon.

**Fig. 5:**
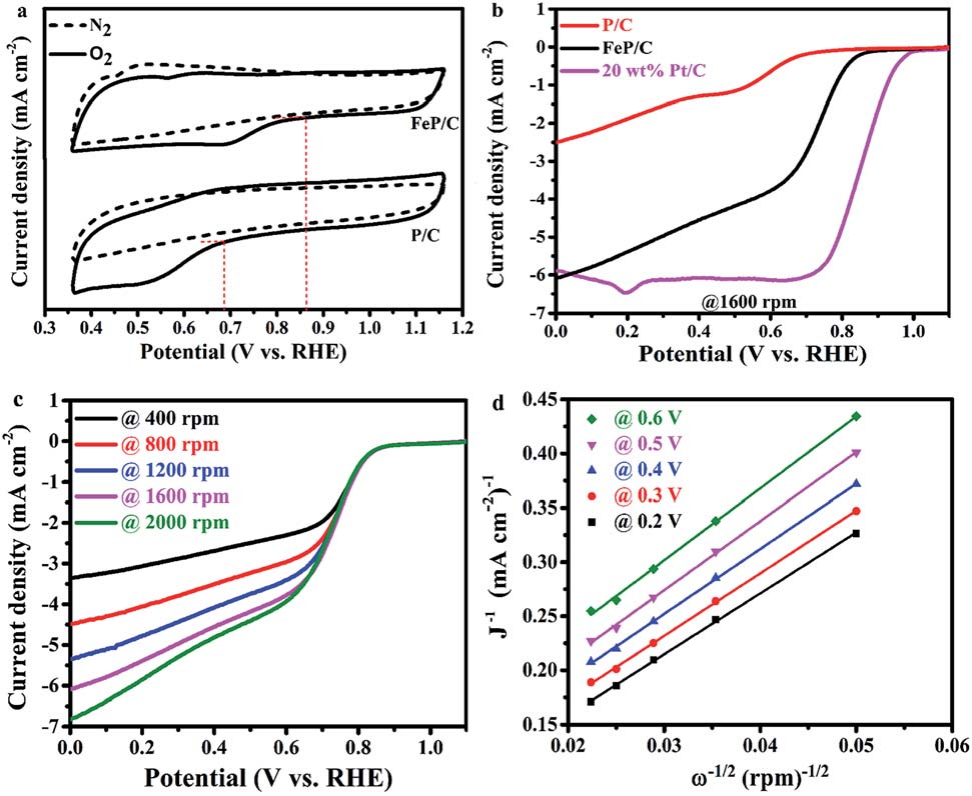
ORR measurements in a three-electrode system using 0.1 M KOH as an electrolyte. (a) CV curve comparison of P/C and FeP/C at 100 mV s^−1^, (b) LSV curve comparison of P/C, FeP/C, and 20 wt% Pt/C at 1600 rpm, (c) LSV curves of FeP/C at different rotation rates, and (d) Koutecky–Levich plots at different potentials.

The electrochemical activities of the as-synthesized samples toward ORR were studied in O_2_- and N_2_-saturated 0.1 M KOH solutions by cyclic voltammetry (CV) at a scan rate of 100 mV s^−1^ (Fig. 5a and S6†). It is clear in Fig. 5a that the CV curves in the N_2_-saturated solution did not display any characteristic reduction peaks, while the same electrodes in the O_2_-saturated solution display characteristic peaks on the cathodic scan at about 0.52 and 0.68 V *vs.* RHE for P/C and FeP/C, respectively. Moreover, as shown in Fig. S6,† the area under the FeP/C curve is clearly higher than that of P/C, indicating the increase in ORR active sites with the introduction of Fe. The ORR curves of FeP/C are well defined with the most positive onset potential (*ca.* 0.86 V *vs.* RHE), which is 180 and 20 mV more positive than those of P/C (Fig. 5a) and a previously reported Fe–phosphate/C,^41^ respectively.

The ORR kinetics of P/C and FeP/C have been studied by linear sweep voltammetry (LSV) through RRDE measurements at rotation speeds ranging from 400 to 2000 rpm with a scan rate of 10 mV s^−1^ (Fig. 5b and c). The LSV curves of as-synthesized FeP/C show a more positive value of onset potential (0.86 V *vs.* RHE) than that of prepared P/C (0.68 V *vs.* RHE), as shown in Fig. 5b. Also, the half-wave potential values of FeP/C, P/C, and commercial Pt/C are estimated to be 0.74 V, 0.59 V, and 0.86 V (*vs.* RHE), respectively. These results indicate the better ORR catalytic activity of prepared FeP/C than that of P/C sample in 0.1 M KOH solution. Interestingly, the limiting current density of our FeP/C electrode at 0 V *vs.* RHE was a little bit higher than that of the commercial Pt/C electrode. Fig. S7† shows the differences in the potential and limiting current density values of the three electrodes. The higher ORR activity of FeP/C than P/C may be due to the higher surface area, the presence of sufficient P–C interactions which break the electroneutrality of carbon, and the presence of Fe atom which helps to break the O=O bonds.^51–55^

Using the Koutecky–Levich plot, the electrons transferred per O_2_ molecule (*n*) during ORR at different potentials (0.2, 0.3, 0.4, 0.5, and 0.6 V *vs.* RHE) were calculated, as shown in Fig. 5d. The obtained K–L plots show high linearity, suggesting first-order reaction kinetics based on our measurement conditions.^56^ The average electron number is 4.6 for FeP/C, which reveals the preferred H_2_O formation mechanism.^36,57^

**Fig. 6:**
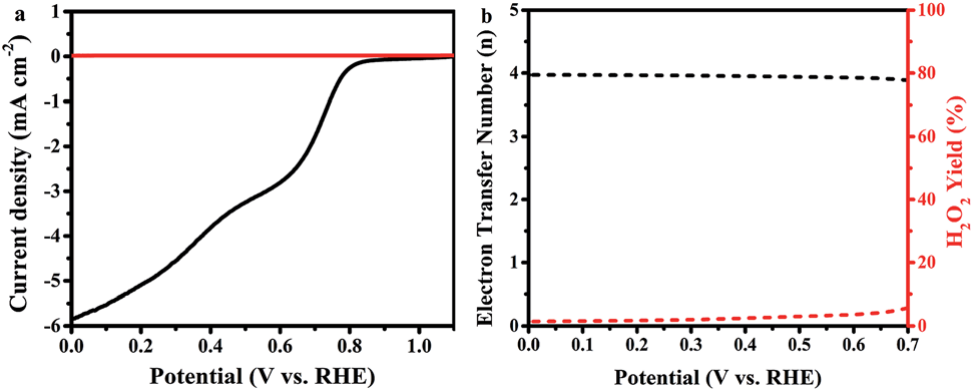
(a) RRDE test of the ORR on FeP/C in an O_2_-saturated 0.1 M KOH electrolyte at a scan rate of 10 mV s^−1^ and a rotating rate of 1600 rpm; (b) assessment of electron transfer number and peroxide percentage in ORR catalyzed by FeP/C based on RRDE data.

In further study, the electron transfer numbers and hydrogen peroxide percentage were calculated by using the currents obtained from disk and ring electrodes through RRDE measurement in the potential range of 0.0–0.7 V *vs.* RHE (Fig. 6).^37^ As shown in Fig. 6b, electron transfer number values are around 4 along all selected potential ranges, which is consistent with the results obtained by K–L plot analysis. The hydrogen peroxide yield is between 1.4% and 5.7%, which agrees with a previously reported study of Fe–phosphate/C material,^41^ which overcomes the reported defects of phosphate-based ORR electrocatalysts.^58^ Such a high electron transfer number and low H_2_O_2_ percentage are very important, according to the operating point of view that requires protection of the catalyst layer and membrane from degradation by crossover H_2_O_2_.^59^

The stability of the FeP/C electrode was investigated using chronopotentiometry measurements at a constant potential (0.46 V *vs.* RHE) in O_2_-saturated 0.1 M KOH at 1600 rpm as a rotation speed and compared with the benchmark Pt/C catalyst (Fig. S8†). The as-synthesized FeP/C electrode retains 84% of its initial current value after 9000 seconds, a bit better than 20 wt% Pt/C, which shows 81% current retention under the same measurement conditions. All of the above results highlight the promising application of FeP/C as an electrode for ORR applications.

## Conclusions

In summary, we have successfully synthesized FeP/C material using an eco-friendly and simple hydrothermal method followed by carbonization. FeP/C sample shows a high surface area (1269 m^2^ g^−1^) that is mainly microporous with the presence of mesopores. FeP/C has been also investigated as an electrode for supercapacitor and ORR applications using a three-electrode system. The electrochemical properties of P/C are significantly improved by incorporating Fe. When FeP/C is used as a supercapacitor electrode, it exhibits a maximum areal capacitance of 313 mF cm^−2^ at a current density of 1.2 mA cm^−2^, accompanied by 95% capacitance retention after 10 000 cycles. While in use as an electrode material for ORR in an alkaline medium, it illustrated high electrocatalytic activity and exhibited a positive shift value of onset potential (0.86 V *vs.* RHE) and high limiting current at 0 V *vs*. RHE with the production of a low percentage from hydrogen peroxide. It also provided a four-electron transfer pathway with 84% current retention after 9000 seconds. The reported results would provide useful information for the development of metal phosphide based electrodes with high performance for supercapacitor and ORR applications.

## Conflicts of interest

There are no conflicts to declare.

## Supplementary Material

RA-009-C9RA04326H-s001
