# Supplementary material for: Iron phosphide anchored nanoporous carbon as an efficient electrode for supercapacitors and the oxygen reduction reaction
Source: RSC Adv. 2019 Aug 13;9(43):25240–7. doi: 10.1039/c9ra04326h (PMC9070042; doi:10.1039/c9ra04326h)
Supplement: RA-009-C9RA04326H-s001 [file RA-009-C9RA04326H-s001.pdf]

Electronic Supplementary Material (ESI) for RSC Advances.
This journal is © The Royal Society of Chemistry 2019

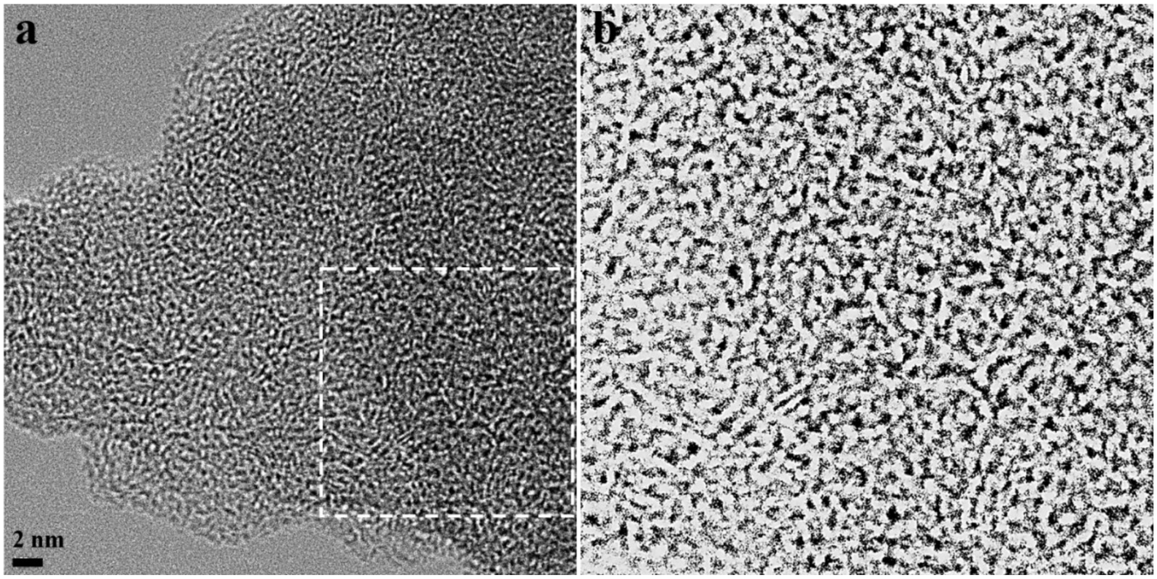

Fig. S1. (a) Low- and (b) high-magnification HRTEM images of prepared FeP/C catalysts.

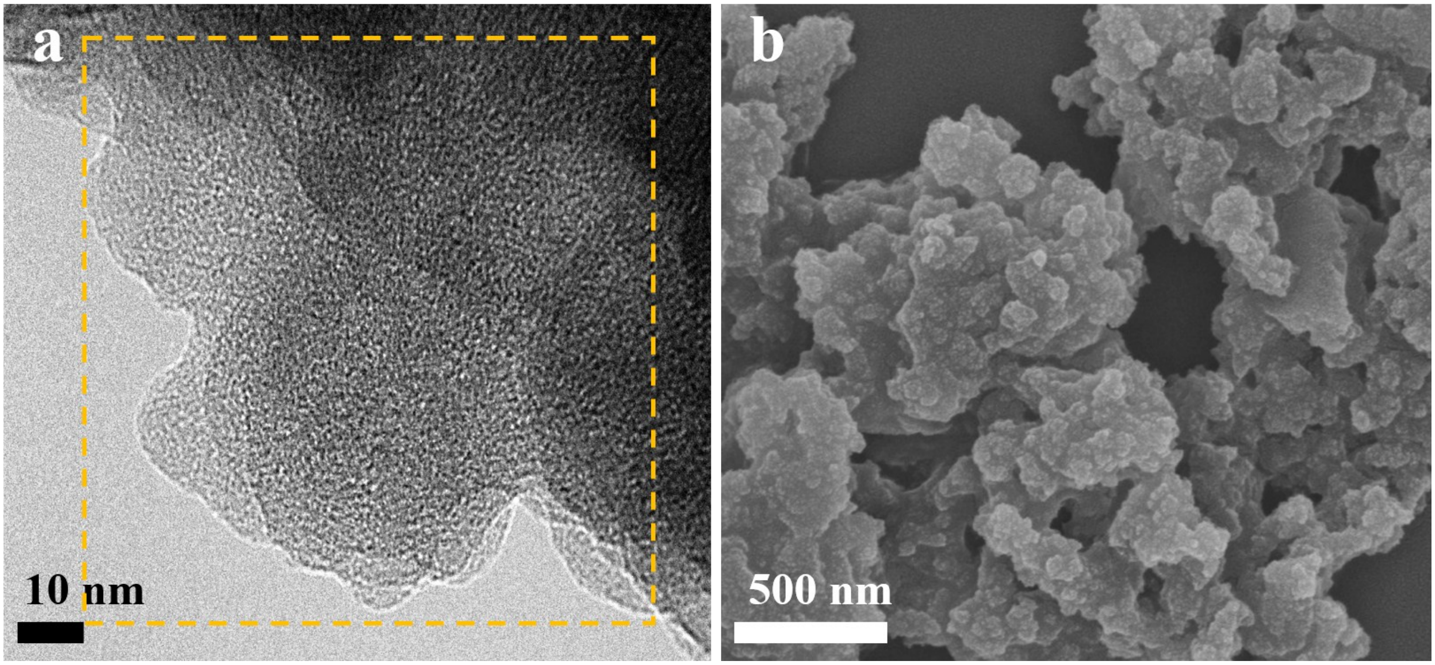

Fig. S2. (a) HRTEM and (b) SEM images of prepared FeP/C samples. The yellow box indicates the layered-like structure of FeP/C.

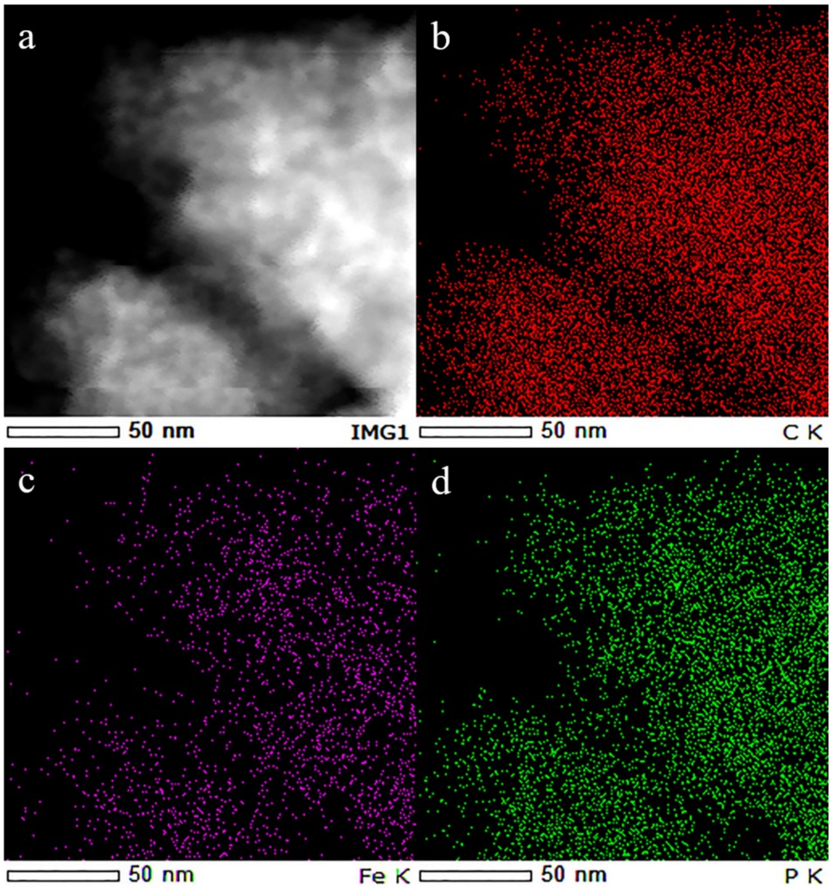

Fig. S3. (a) HAADF-STEM and corresponding (b–d) HAADF-STEM-EDS elemental mapping images of C, Fe, and P.

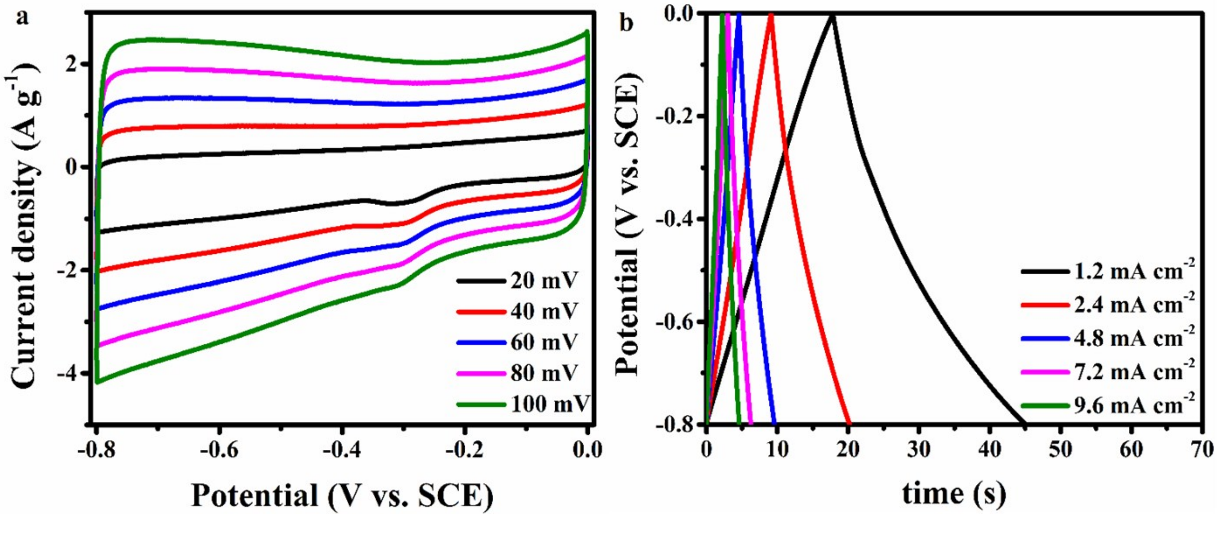

Fig. S4. (a) CV curves of P/C at various scan rates; (b) GCD curves of P/C at different current densities.

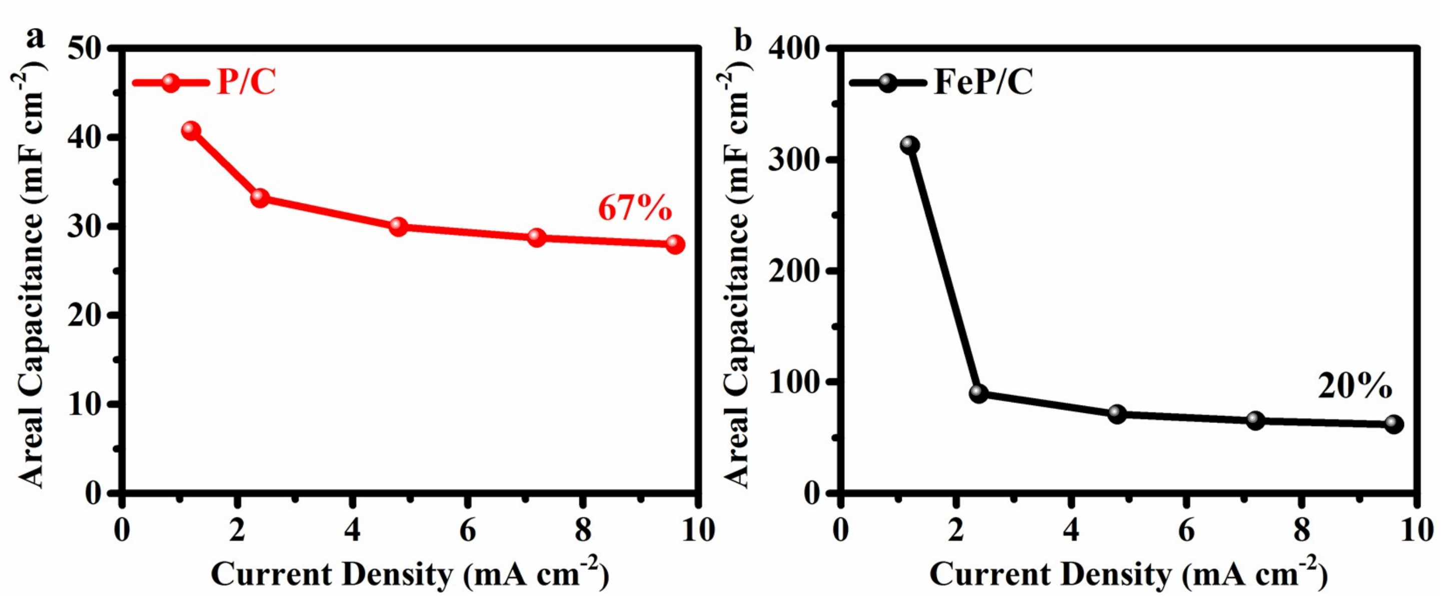

Fig. S5. Areal capacitances at different current densities of (a) P/C and (b) FeP/C samples.

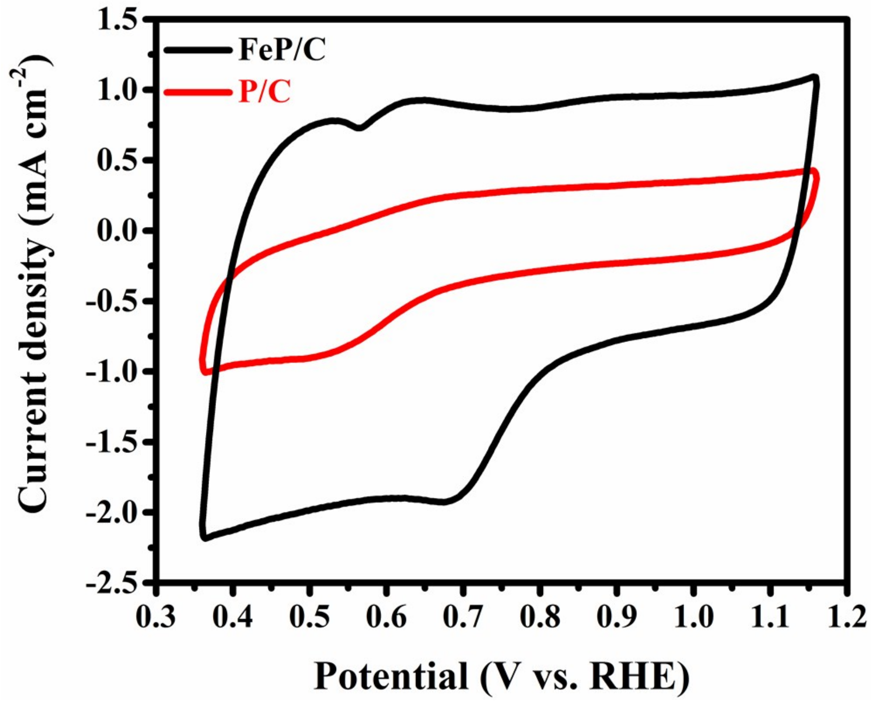

Fig. S6. Cyclic voltammetry curve for P/C and FeP/C recorded in O_2-saturared 0.1 M KOH at a scan rate of 100 mV s^{-1}.

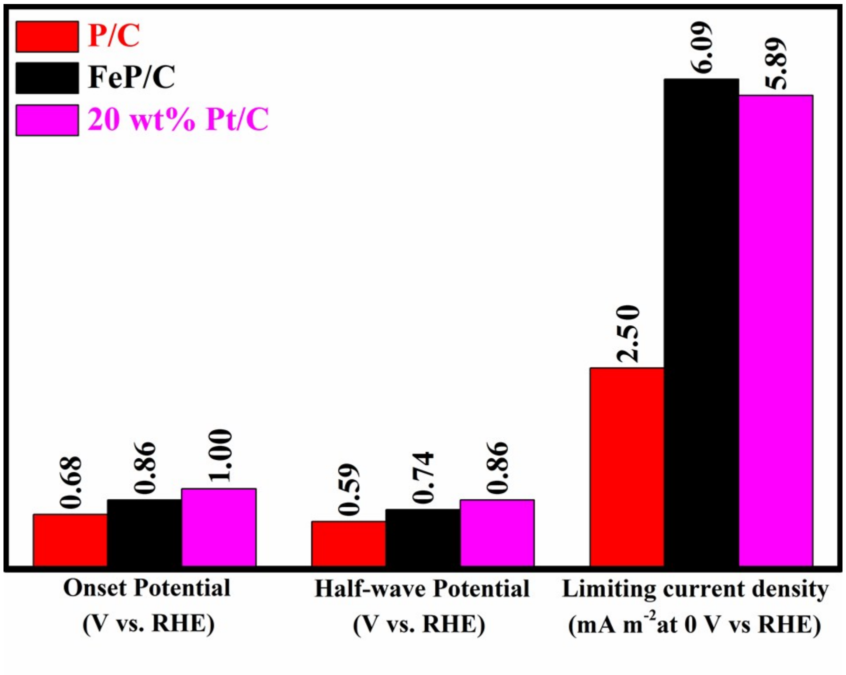

Fig. S7. Comparison of the onset, half-wave potentials, and limiting current densities of P/C, FeP/C, and 20 wt% Pt/C.

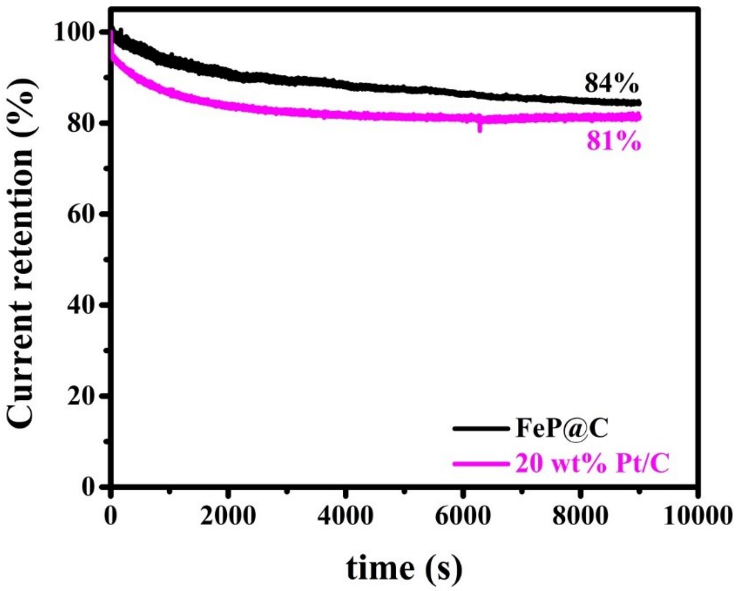

Fig. S8. Chronoamperometric measurements of FeP/C and commercial 20 wt% Pt/C for 9000 s at a constant potential of 0.46 V *vs.* RHE.

Table S1. The specific surface area, pore width, and total pore volume of P/C and FeP/C.

Sample	Pore size (nm)	Specific surface area ($m^2\ g^{-1}$)	Total pore volume ($cm^3\ g^{-1}$)
P/C	1.79	596	0.69
FeP/C	0.67	1269	1.44

Table S2. Comparison data for the electrochemical performance of FeP/C with previously reported Fe-based materials.

Electrode material	Areal capacitance ($mF\ cm^{-2}$)	Current density ($mA\ cm^{-2}$)	Electrolyte	Electrode stability	Ref.
FeP/C	313	1.2	3 M KOH	95% at 10,000 cycles 100 mV s^{-1}	This work
FeP nanotube arrays	300.1	1	1 M LiCl	41% at 5000 cycles 5 mA cm^{-2}	[1]
$Fe_2P_4O_{12}$–carbon composite	251	1	0.5 M H_2SO_4	Initial 10% loss after 500 cycles 5 mA cm^{-2}	[2]
Fe_2O_3 nanotubes	180.4	1	5 M LiCl	84% at 5000 cycles 2 mA cm^{-2} (in asymmetric supercapacitor)	[3]
C@Fe_3O_4 yarn	127	1	1 M LiCl	80% at 800 cycles (in solid-state asymmetric supercapacitor)	[4]

Fe_2O_3 nanoflake	145.9	1	3 M LiCl	87.2% at 5000 cycles	[5]

References

1. B. Liang, Z. Zheng, M. Retana, K. Lu, T. Wood, Y. Ai, X. Zu and W. Zhou, *Nanotechnology*, 2019, **30**, 295401.

2. R. Soni and S. Kurungot, *New J. Chem.*, 2019, **43**, 399-406.

3. P. Yang, Y. Ding, Z. Lin, Z. Chen, Y. Li, P. Qiang, M. Ebrahimi, W. Mai, C. P. Wong and Z. L. Wang, *Nano Lett.*, 2014, **14**, 731-736.

4. J. Sun, Y. Huang, C. Fu, Y. Huang, M. Zhu, X. Tao, C. Zhi and H. Hu, *J. Mater. Chem. A*, 2016, **4**, 14877-14883.

5. T. Liu, Y. Ling, Y. Yang, L. Finn, E. Collazo, T. Zhai, Y. Tong and Y. Li, *Nano Energy*, 2015, **12**, 169-177.
